# Medical Schools

**Published:** 1846-02

**Authors:** 


### MEDICAL INTELLIGENCE.

### MEDICAL SCHOOLS.

We have received the catalogue and circular of the following Institutions for the winter session of 1845-46.

Louisville Medical Institute. Number of Students who Matriculated, 345.

Willoughby Medical College. Number of Matriculants 164. Number of Graduates, 30.

Albany Medical College. Number of Students, 115.

Vermont Medical College, at Woodstock, (Announcement.)

Western Reserve College, Medical Department at Cleveland, Ohio. Number of Students, 161.

---

*Rush Medical College.*—The Degree of Doctor of Medicine was conferred upon the following young gentlemen at the annual commencement of the Rush Medical College, February 19, 1846.

| Graduates of 1846. | | Subject of Thesis. |
|---|---|---|
| Elwood Andrew, | Indiana, | Therapeutic virtues of the Euonymus Atropurpureus. (Wahoo.) |
| J. Herman Bird, | New York, | Carcinoma. |
| Daniel K. Hays, | Indiana, | Duties of young Graduates in Medicine. |
| James M. Higby, | Michigan, | Quinia. |
| Newton P. Holden, | Illinois, | Dyspepsia. |
| Alex. Malcolm, | Iowa Ter. | Fever. |
| Cicero Robbe, | Illinois, | Duties of the Student and Practical Physician. |
| Halsey Rosenkrans, | Illinois, | Auscultation. |
| Wm. W. Welch, | New York, | Contagion. |

The honorary Degree of Doctor of Medicine was conferred on Wm. G. Montgomery, of Warren county, Indiana.

